# An integrated multigene expression panel to predict long-term survival after curative hepatectomy in patients with hepatocellular carcinoma

**DOI:** 10.18632/oncotarget.20369

**Published:** 2017-08-19

**Authors:** Mitsuro Kanda, Kenta Murotani, Hiroyuki Sugimoto, Takashi Miwa, Shinichi Umeda, Masaya Suenaga, Masamichi Hayashi, Norifumi Hattori, Chie Tanaka, Daisuke Kobayashi, Suguru Yamada, Michitaka Fujiwara, Yasuhiro Kodera

**Affiliations:** ^1^ Department of Gastroenterological Surgery (Surgery II), Nagoya University Graduate School of Medicine, Nagoya, Japan; ^2^ Clinical Research Center, Aichi Medical University Hospital, Nagakute, Japan

**Keywords:** hepatocellular carcinoma, biomarker, prognosis, expression panel

## Abstract

Hepatocellular carcinoma (HCC) frequently recurs even after curative hepatectomy. To develop an integrated multigene expression panel, 144 patients were randomly assigned to either discovery or validation set in a 1:2 ratio. Using surgically resected HCC specimens, expression levels of 12 candidate molecular markers were determined using quantitative reverse-transcriptase PCR. In the discovery set, an expression panel was developed according to the concordance index (C-index) values for overall survival from all 4095 combinations of the 12 candidate molecular markers. Expression scores was determined with weighting according to the coefficient in a Cox regression, and patients were classified into grade 1, 2 and 3. Reproducibility was then tested in the validation set. A panel consisting of four markers, *PRMT5*, *MAGED4*, *DPYSL3* and *AJAP1* was selected as the optimal and most well-balanced set with a C-index value of 0.707. Patient prognosis was clearly stratified by the expression grade using this panel. In the validation set, both overall and disease-free survival rates decreased incrementally with as the grade increased. Higher grades were significantly associated with tumor multiplicity and vessel invasion. The prevalence of extrahepatic recurrences was increased in grade 3 patients. The integrated multigene expression panel clearly stratified HCC patients into low, intermediate and high risk.

## INTRODUCTION

Hepatocellular carcinoma (HCC) represents a major health problem, with the third highest mortality rate among all malignancies worldwide [1]. Because HCC is characterized by its extensive clinical heterogeneity, accurate survival risk stratification is a critical clinical task in the management and treatment of this disease [2, 3]. While referring to the TNM classification has long been the standard in risk stratification, the current version does not contain clues for predicting differences in biology of the individual tumors and, ultimately, the outcome so as to promote precision medicine [3, 4].

To date, numerous molecular biomarkers have been reported as indicators for early detection, prognosis, patterns of disease recurrence and treatment response [5]. Although some individual markers were found to be promising and attractive, single markers have inherent limitations in sensitivity, specificity, and accuracy in risk stratifications and are less likely to reflect the diverse tumor microenvironment [6, 7]. Recently, panels of multiple markers have been proposed to overcome these shortcomings and maximize their clinical utility, and have shown successful results in other types of cancer [8, 9]. Identification of patients who are expected to have excellent long-term outcomes contributes to the healthcare system by avoiding unnecessary imaging and clinical examinations. On the contrary, identification of patients at high risk of recurrence and with an adverse prognosis is helpful for physicians in decision-making, enabling them to select patients eligible for intensive follow-up and treatment intervention.

Cumulatively, 12 molecular markers for HCC including cancer-specific antigens, tumor suppressors and cell adherents have been discovered at Nagoya University since 2013. The aim of this study was to test the hypothesis that predictive performance can be improved by developing a multigene expression panel consisting of 12 original molecular markers and aid in the selection of a sensitive risk stratification protocol in patients with resectable HCC.

## RESULTS

### Development of an integrated multigene expression panel

**Figure 1:**
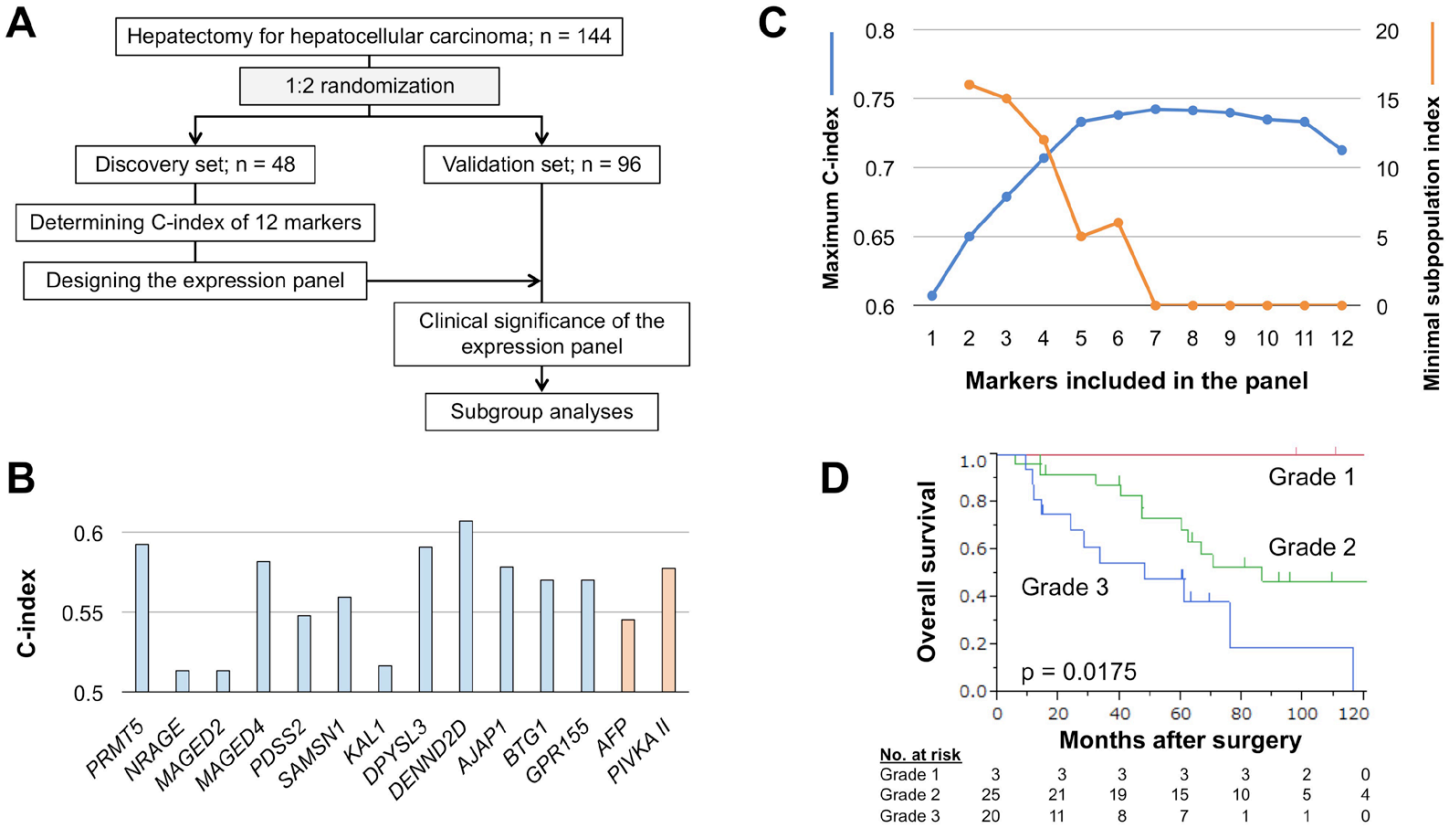
Development of the integrated multigene expression panel **(A)** Study flowchart. **(B)** C-index values of the 12 candidate molecular markers and preoperative serum AFP and PIVKA-II. **(C)** Changes in C-index values and the subpopulation index according to the number of constituents. **(D)** Overall survival of patients in expression grades 1, 2 and 3.

Study design was summarized in Figure 1A. There were no significant differences in demographics, background liver status, hepatic virus infection, tumor multiplicity, tumor size, serosal infiltration, vascular invasion and disease stage between the discovery (n = 48) and validation (n = 96) sets (Supplementary Table 1). Patients in the discovery and validation set were followed up for median 66.3 and 83.9 months, respectively, or until death.

The expression panel was designed using the discovery set. C-index values of single candidate markers ranged from 0.513–0.607, and those of preoperative serum alpha-fetoprotein (AFP; cutoff 20 ng/ml) and protein induced by vitamin K antagonists (PIVKA)-II (cutoff 40 mAU/ml) were 0.545 and 0.577, respectively (Figure 1B). C-index values of all single and combinations of 12 candidate markers (neither AFP nor PIVKA-II included) for overall survival were calculated and counted for 4095 patterns. The highest C-index value among all combinations was 0.742, which was determined for the expression panel consisting of seven markers (Figure 1C). The larger the number of markers included in the panel, the greater the number of subpopulations that patients were clustered into with a corresponding decrease in the minimal number of patients in a subpopulation. The subpopulation index, number of constituents × the minimal patient number in a subpopulation, rapidly decreased after the number of markers was ≥5 (Figure 1C). Accordingly, the optimal and balanced number of markers was determined as four (C-index >0.7 and subpopulation index >10).

The panel having the greatest C-index among combinations of four constituents consisted of *PRMT5*, *MAGED4*, *DPYSL3* and *AJAP1*, and the C-index value was 0.707 (Supplementary Table 2). After weighting each marker using the coefficient, expression scores were determined for all 48 patients, and then provisionally graded into grade 1 (expression score 0–50), grade 2 (score 51–150) and grade 3 (score ≥151). As this grading system clearly stratified patients having favorable, moderate and poor overall survival (Figure 1D), it proceeded to the validation stage.

### Validation of predictive performance of the integrated multigene expression panel

**Figure 2:**
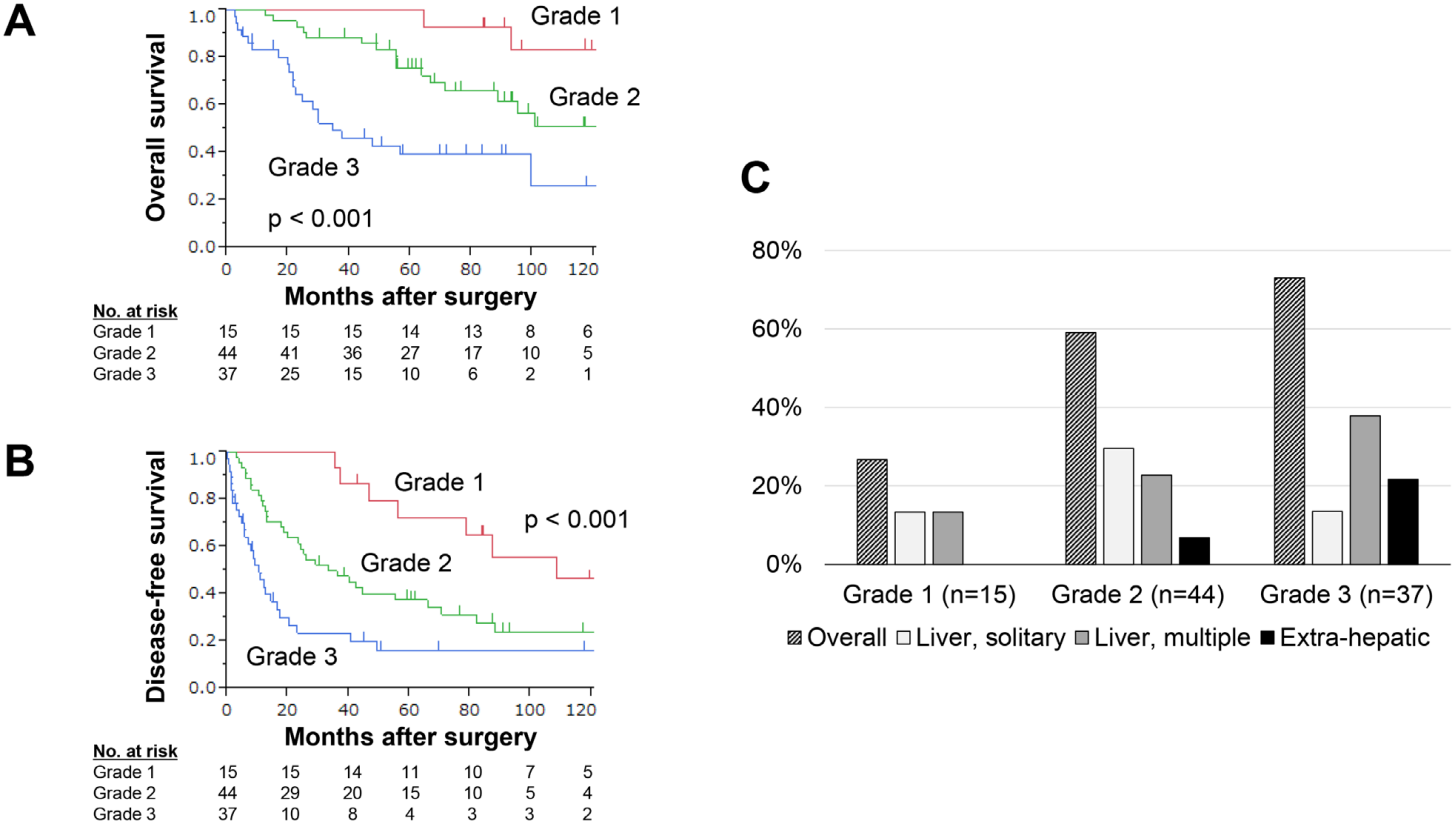
Performance of the integrated multigene expression panel in the validation set **(A)** Overall survival of patients in expression grades 1, 2 and 3. **(B)** Disease-free survival of patients in the expression grades 1, 2 and 3. **(C)** Overall recurrence rates and frequency of each recurrent pattern according to expression grade.

The reproducibility of the expression panel was evaluated using the validation cohort (n = 96). With respect to overall survival, the prognosis of patients in grades 1, 2 and 3 were clearly distinguished from each other (Figure 2A). Similarly, disease-free survival rates gradually decreased with increasing grade (Figure 2B). These results demonstrated that the integrated multigene expression panel could clearly stratify patients into low, intermediate and high risk for long-term survival after hepatectomy. Moreover, multivariable analysis identified expression grade 3 as an independent prognostic factor for overall survival (hazard ratio 2.12, 95 % confidence interval 1.12 – 4.04, *P* = 0.003; Supplementary Table 3). The prognostic value of single markers, the four constituents of the expression panel (*PRMT5*, *MAGED4*, *DPYSL3* and *AJAP1*) and the preoperative serum markers AFP and PIVKA-II in the validation set are shown in Supplementary Figure 1. None of them exhibited the equivalent stratifying performance compared with the multigene expression panel.

### Comparison of clinical characteristics for each grade

Next, the association between the grade and clinicopathological parameters was evaluated. No significant differences were found with respect to age, sex or background liver status. Conversely, higher grade was significantly associated with elevated preoperative AFP levels, tumor multiplicity, vessel invasion and advanced disease stage (Table 1). The recurrence patterns were classified into a solitary recurrence confined to the liver, multiple recurrences confined to the liver confined, and extrahepatic (distant) recurrences. Overall recurrence rates and frequency of the three types of recurrences observed for each expression grade are depicted in Figure 2C. No grade 1 patients experienced extrahepatic recurrences, whereas the proportion of multiple liver and extrahepatic recurrences was increased in grade 3 patients (Figure 2C).

**Table 1:** Association between expression grade and clinicopathological parameters in the validation set

	Grade 1	Grade 2	Grade 3	*P*
Age				0.514
< 65 year	5	22	16	
≥ 65 year	10	22	21	
Sex				0.468
Male	12	40	31	
Female	3	4	6	
Background liver				0.444
Normal liver	1	4	3	
Chronic hepatitis	6	28	19	
Cirrhosis	8	12	15	
Pugh-Child’s classification				0.533
A	13	42	34	
B	2	2	3	
Hepatitis virus				0.928
Absent	2	9	7	
HBV	3	11	10	
HCV	10	24	20	
AFP (ng/ml)				<0.001
≤ 20	10	31	9	
> 20	5	13	28	
PIVKA II (mAU/ml)				0.427
≤ 40	8	20	13	
> 40	7	24	24	
Tumor multiplicity				0.031
Solitary	14	37	24	
Multiple	1	7	13	
Tumor size				0.069
< 3.0 cm	6	20	8	
≥ 3.0 cm	9	24	29	
Differentiation				0.497
Well	3	13	7	
Moderate to poor	12	31	30	
Growth type				0.594
Expansive growth	13	38	29	
Invasive growth	2	6	8	
Pathological serosal infiltration				0.418
Absent	12	35	25	
Present	3	9	12	
Pathological vascular invasion				<0.001
Absent	12	39	18	
Present	3	5	19	
UICC pathological stage				<0.001
I	11	35	14	
II	3	8	12	
III	1	1	11	

HBV, hepatitis B virus; HCV, hepatitis C virus; AFP, alpha-fetoprotein; PIVKA, protein induced by vitamin K antagonists; UICC, Union for International Cancer Control.

### Subgroup analyses according to disease stage and background liver status

**Figure 3:**
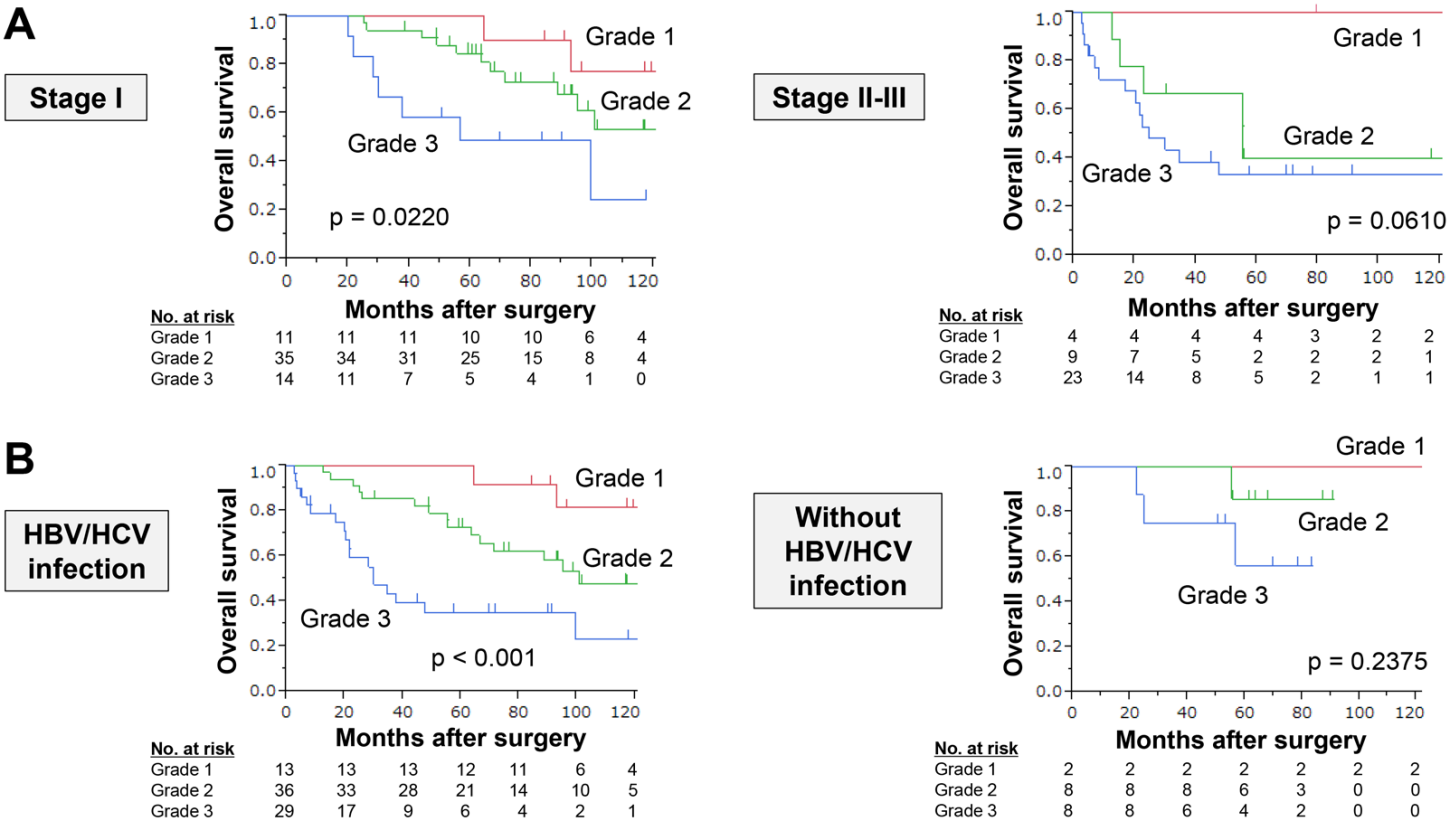
Subgroup analysis **(A)** Overall survival rates in patient subgroups according to disease stage. **(B)** Overall survival rates in patient subgroups according to hepatitis virus infection.

To further evaluate the clinical utility of the expression panel, subgroup analyses of prognostic relevance of the expression grades were conducted according to disease stage (stage I or II/III) and hepatitis virus infection. Either in the subpopulation of stage I or stage II/III, overall survival was clearly distinguishable by the expression grade (Figure 3A). Moreover, overall survival rates were decreased with higher expression grade in both patient subgroups with and without hepatitis B/C infection (Figure 3B).

## DISCUSSION

Here, we describe an integrated multigene expression panel that can stratify patients into low, intermediate and high risk after curative hepatectomy for HCC. The strength of the panel is manifested in multiple ways: it is a novel panel consisting of original molecular markers; it has both predictive value and clinical compatibility; it has confirmed reproducibility as demonstrated in the discovery and validation patient sets.

The C-index value was calculated for all combinations of the 12 molecular markers and several patterns with a C-index value ≥0.7 were identified. The population index was used to optimize the number of markers included in the panel, and inclusion of four markers was found to be the most objectively balanced system. Additionally, scoring and grading were important processes used in determining the performance of the expression panel. A weighting using the coefficient of each constituent was employed to determine the expression score for each patient. Thereafter, patients were divided into three grades according to the expression score, which was a more straightforward patient stratification method compared with using continuous numeric variables. Because these attempts were certainly exploratory and challenging, the validation process was necessary to evaluate the validity of the procedure used in the development of the multigene expression panel. The predictive value of the panel was reproduced successfully in the validation set.

HCC is a complex disease with multiple underlying pathogenic mechanisms including epigenetic modifications, epithelial-mesenchymal transition, cancer microenvironment, apoptosis and chemoresistance [10–12]. These pathogenic mechanisms are complexly intertwined and give rise to the various cancer phenotypes and finally the clinical course of the disease [6, 13]. Thus, it is unlikely that a single molecular marker can faithfully represent the various oncological signatures. Currently, several multigene expression assays (e.g. Oncotype Dx^®^ and MammaPrint^®^) have been commercially available to predict prognosis and evaluate whether adjuvant chemotherapy is appropriate, and to contribute to decision-making in the clinical practice of breast, prostate and colorectal cancers [14–17]. Considering that no multigene assay for HCC has been established for clinical use, accumulating HCC-related molecular data and developing a high-performance multigene panel are needed. We have developed a novel integrated multigene expression panel composed of original markers, and it is expected that incorporation of other known HCC-related markers might further improve the performance of our expression panel. The four constituents of the expression panel (*PRMT5*, *MAGED4*, *DPYSL3* and *AJAP1*) have individual roles in HCC progression as we reported previously [18–21]. These different types of markers complementarily interacted with each other and contributes to the expression panel having an improved predictive performance. At the same time, our results also indicated anew that diverse molecular mechanisms are complexly intertwined and contribute to progression of HCC.

Further examinations will be required to translate results of the present study to the clinic and to determine how best to use the expression panel. Indeed, the single use of our 12 candidate markers showed modest predictive performance as well as AFP or PIVKA-II, which are currently used as tumor markers in HCC [19, 22, 23]. However, our integrated multigene expression panel enables physicians to easily identify individuals expected to have an excellent prognosis (low risk), and conversely those expected to have a dismal prognosis (high risk) immediately after surgery. For patients at low risk, avoidance of excessive intervention both in monitoring and treatment can reduce patient burden and medical cost [3]. On the contrary, intensive systemic surveillance including the chest and pelvic cavity could be considered for patients at high risk in anticipation of early or extra-hepatic recurrences. For patients at intermediate risk, a standard management conformable to the treatment guidelines is recommended.

Another important finding of the present study is that expression grades determined in initially resected HCC tissues were associated with not only the probability but also the patterns of future recurrences. The expression grade gradually increased from 1 to 2 to 3 in patients with solitary intrahepatic recurrences, multiple intrahepatic recurrence, and extrahepatic recurrence, respectively. Accordingly, the expression panel would help physicians to provide appropriate postoperative management including disease monitoring and focusing on systemic metastasis. Moreover, it might merit inclusion as a criterion for prospective clinical trials evaluating survival benefit of systemic adjuvant chemotherapy in HCC. In the current study, expression levels of the molecular markers were determined using surgically-resected liver tissues. As liver biopsy samples are also available for mRNA analysis, expression grades can be determined before surgery and may contribute to decision-making regarding surgical indication and procedure. Data of immunohistochemical staining is important for biomarker studies, particularly towards the clinical applications. We previously evaluated expression of PRMT5, MAGED4, DPYSL3 and AJAP1 proteins in HCC by immunostaining and found that the expression pattern of the proteins correlated with that of mRNA [18, 19, 23, 24].

Limitations of this study include its retrospective nature, usage of some old samples and small cohort size. The present study is also limited because of the long period of study at 14 years, which may have biased the data. Despite an effort to reduce selection bias using a 2-step evaluation, additional validation of the expression panel performance is required. Future large-scale prospective studies are still required for optimization of cutoff values and widespread application of the expression panel in the clinic. Although mRNA expression levels were used because it is easy to quantitatively and objectively measure RNA levels, the use of IHC could be considered given that it is a readily accessible and commonly used technique in clinical practice. Nevertheless, this study concept can leverage current knowledge of single molecular markers and bring it to the next stage, which would be an important step forward in the realization of precision surgery.

In conclusion, the integrated multigene expression panel consisting of original molecular markers was developed for risk stratification of patients with resectable HCC. This concept can be expected to maximize the predictive performance of each single marker, enable clear risk stratification and eventually contribute to personalized medicine in the field of surgical oncology.

## MATERIALS AND METHODS

### Ethics

This study conformed to the ethical guidelines of the Declaration of Helsinki and has been approved by the Institutional Review Board of Nagoya University, Japan. Written informed consent for usage of clinical samples and data, as required by the institutional review board, was obtained from all patients.

### Patients and sample collection

Primary HCC tissues and corresponding noncancerous tissues were collected from 144 patients who underwent curative hepatectomy for HCC at Nagoya University Hospital between January 1998 and January 2012. Treatment after recurrence generally included the following options: surgery, radiofrequency ablation, transcatheter arterial chemoembolization and chemotherapy according to tumor status and liver function. Tissue samples were collected, frozen immediately in liquid nitrogen and stored at –80°C until used for RNA extraction (average 28 days). RNA was extracted from tumor samples with approximately 5 mm diameters that did not contain a necrotic component. The tumor was pathologically diagnosed as HCC, and the area containing more than 80% of the cancer cells was selected for RNA extraction. Using a table of random numbers, 144 patients were divided into discovery (n = 48) and validation sets (n = 96) in a 1:2 ratio. Markers to be included in the integrated expression panel were determined using the discovery set, and the clinical predictive performance of the panel was subsequently evaluated in the validation set. We employed 1:2 allocation because the second patient set having a sample size as large as possible is needed to validate the clinical significance of the expression panel more reliably in a correlation analysis with clinicopathological factors and recurrence patterns.

### Candidate molecular markers and measurement of mRNA expression levels

In this study, 12 candidate markers were selected from our recently published data from biomarker studies in HCC (Table 2). The following biomarkers were subjected to expression analysis: Protein arginine methyltransferase 5 (*PRMT5*), neurotrophin receptor-interacting melanoma antigen-encoding protein (*NRAGE*), melanoma antigen gene family member D2 and D4 (*MAGED2* and *MAGED4*), decaprenyl diphosphate synthase subunit 2 (*PDSS2*), SAM domain, SH3 domain and nuclear localization signals 1 (*SAMSN1*), Kallmann syndrome 1 gene (*KAL1*), dihydropyrimidinase-like 3 (*DPYSL3*), DENN domain containing 2D (*DENND2D*), adherens junctions associated protein 1 (*AJAP1*), BTG anti-proliferation factor 1 (*BTG1*), and G protein-coupled receptor 155 (*GPR155*) [18, 19, 22–31].

**Table 2:** List of candidate markers aberrantly expressed in hepatocellular carcinoma

Symbol	Name	Location	Function	Status in HCC*	Cutoff*
*PRMT5*	protein arginine methyltransferase 5	14q11.2	Transcriptional regulation, and the assembly of small nuclear ribonucleoproteins	Upregulated	C median
*NRAGE*	Neurotrophin receptor-interacting melanoma antigen-encoding protein	Xp11.22	Pro-apoptotic factor required for the normal developmental apoptosis	Upregulated	C/N >1
*MAGED2*	MAGE family member D2	Xp11.21	Tumor specific antigens	Upregulated	C/N >1
*MAGED4*	MAGE family member D4	Xp11.22	Tumor specific antigens	Upregulated	C/N >3
*PDSS2*	decaprenyl diphosphate synthase subunit 2	6q21	Synthesis of coenzyme Q10	Downregulated Hypermethylated	C/N <0.5
*SAMSN1*	SAM domain, SH3 domain and nuclear localization signals 1	21q11.2	Cytoplasmic adaptor protein	Downregulated Hypermethylated	C median
*KAL1*	Kallmann syndrome 1	Xp22.31	Neural cell adhesion and axonal migration	Downregulated Hypermethylated	C/N <0.5
*DPYSL3*	dihydropyrimidinase like 3	5q32	Cell-adhesion factor	Downregulated Hypermethylated	C median
*DENND2D*	DENN domain containing 2D	1p13.3	Membrane trafficking protein regulating Rab GTPases	Downregulated Hypermethylated	C/N <0.3
*AJAP1*	adherens junctions associated protein 1	1p36.32	Component of adherens junctions	Downregulated Hypermethylated	C median
*BTG1*	BTG anti-proliferation factor 1	12q21.33	Regulates cell growth and differentiation	Downregulated	C/N <0.4
*GPR155*	G protein-coupled receptor 155	2q31.1	Mediator of the visual sensing, immune function, and cell proliferation	Downregulated	C/N <0.5

*From our previous studies.

Quantitative real-time reverse transcription PCR was performed to determine mRNA expression levels. Total RNA (10 μg per sample) was isolated and used as template for complementary DNA synthesis. A quality check for all RNA samples was conducted before generating complementary DNAs. The optical density was measured and the ratio of the absorbance at 260 and 280 nm ranged from 1.8 to 2.0 in all samples. Primer sequences used in this study are listed in Supplementary Table 4. One hundred and forty-four pairs of liver tissues were analyzed using a SYBR Green PCR Core Reagent Kit (Applied Biosystems, Foster City, CA, USA) that included samples without template as a negative control. An ABI StepOnePlus Real-Time PCR System (Applied Biosystems) was used for detection of SYBR Green fluorescence emission intensity. The expression of glyceraldehyde-3-phosphate dehydrogenase (*GAPDH*) mRNA was quantified in each sample and used to standardize the data. Technical replicates were performed in triplicate for all samples. Expression levels of each sample are shown as the value of each target divided by the *GAPDH* value. Patients were categorized into the two groups using cutoff values from previous studies (Table 2).

### Creation and validation of the integrated multigene expression panel

To design an integrated multigene expression panel, the following processes were carried out using the discovery set. First, concordance index (C-index) values for overall survival were calculated for all 12 candidate markers, both alone and in various combinations with any number of other markers adding up to a total of 4,095 combination patterns, . Second, the best C-index values and minimal sample sizes in a cluster for each number of combinations (1–12) were calculated to determine the optimal number of markers to be included in the expression panel. Third, the expression panel that yielded the highest C-index was proposed. Fourth, the expression score was determined in all 48 patients with weighting according to the coefficient in a Cox regression of each constituent. Fifth, patients were classified into grade 1, 2 or 3 according to the expression score. Provisional cutoff for the grading (grade 1 to 3) were determined in the discovery set based on the following concept. The cutoff line for expression grade 1 was set strictly to achieve careful selection of patients with excellent postoperative outcomes, even if the population becomes small. Similarly, cutoff line for expression grade 3 was set to select patients at very high risk. Last, the reproducibility and predictive performance of the integrated multigene expression panel was tested in the validation set. Subgroup analyses in which the patients were stratified according to TNM stage and background hepatitis virus infection were also conducted.

### Statistical analysis

The qualitative χ^2^ and quantitative Mann–Whitney tests were used to compare the two groups. Survival rates were calculated using the Kaplan–Meier method, and the difference between curves was analyzed using the log-rank test. The Cox regression model was used to evaluate the overall survival hazard ratio associated with each variable and multivariable analysis to detect independent prognostic factors. Variables with *P* < 0.05 were entered into the final model. The prediction score was internally validated by the C-index. The C-index is a probability of concordance between predicted and observed survival, with C = 0.5 for random predictions and C = 1 for a perfectly discriminating score. The C-index was evaluated on the discovery set using bootstrapping with 10,000 resamples [32]. Statistical analysis was performed using JMP 10 software and SAS9.4 (SAS Institute Inc., NC). *P* <0.05 indicates a statistically significant difference.

## SUPPLEMENTARY MATERIALS FIGURES AND TABLES


