# Supplementary material for: An integrated multigene expression panel to predict long-term survival after curative hepatectomy in patients with hepatocellular carcinoma
Source: Oncotarget. 2017 Aug 19;8(41):71070–9. doi: 10.18632/oncotarget.20369 (PMC5642618; doi:10.18632/oncotarget.20369)
Supplement: Supplementary file 1 [file oncotarget-08-71070-s001.pdf]

An integrated multigene expression panel to predict long-term survival after curative hepatectomy in patients with hepatocellular carcinoma

SUPPLEMENTARY MATERIALS

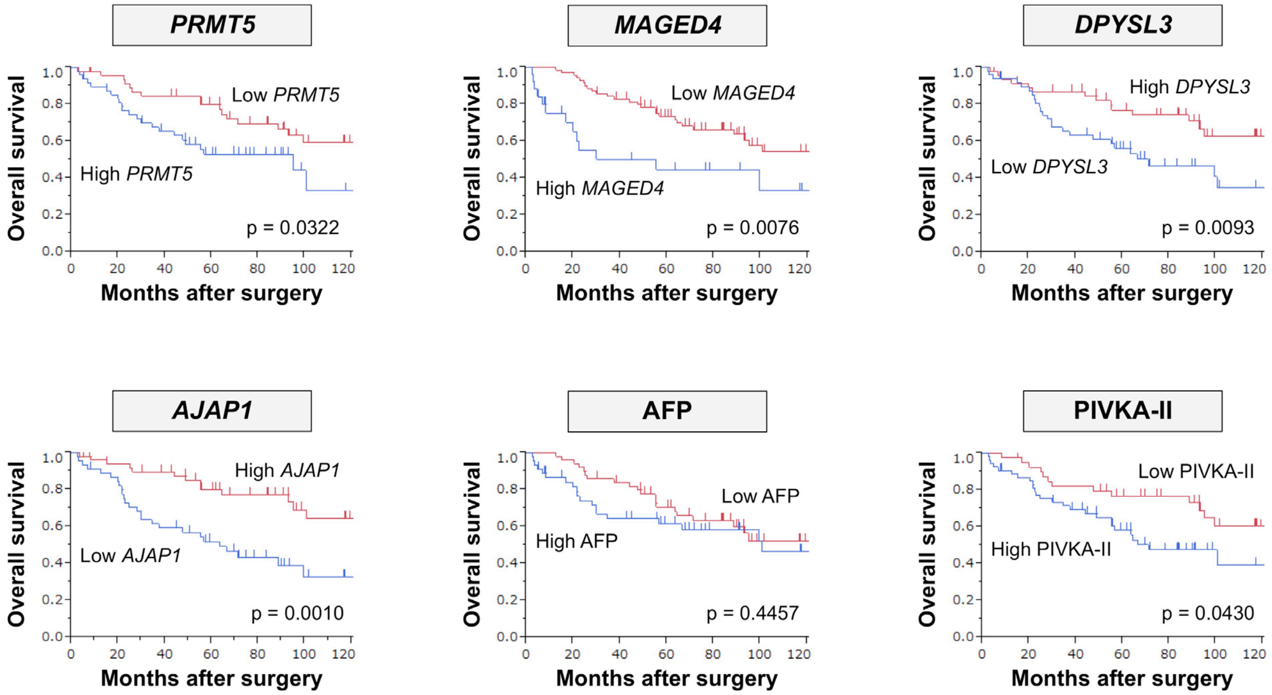

Supplementary Figure 1: The prognostic value of the single markers.

Supplementary Table 1: Characteristics of patients in the discovery and validation sets

	**Discovery set (n=48)**	**Validation set (n=96)**	***P***
Age (years), mean ± SD	64.0 ± 10.0	64.7 ± 9.6	0.837
Sex (male/female)	38 / 10	83 / 13	0.268
Background liver			0.596
Normal liver	2	8	
Chronic hepatitis	29	53	
Cirrhosis	17	35	
Hepatitis virus			0.961
Absent	9	18	
HBV	13	24	
HCV	26	54	
Tumor multiplicity			0.888
Solitary	37	75	
Multiple	11	21	
Tumor size			0.201
< 3.0 cm	12	34	
≥ 3.0 cm	36	62	
Pathological serosal infiltration			0.783
Absent	37	72	
Present	11	24	
Pathological vascular invasion			0.213
Absent	39	69	
Present	9	27	
UICC pathological stage			0.574
I	30	60	
II	14	23	
III	4	13	
Follow up months, median	66.3	83.9	0.230

SD, standard deviation.

Supplementary Table 2: Proposed expression panel to predict overall survival

Symbol	C-index; single marker	C-index; expression panel	Coefficient	HR	95% CI	*P*
PRMT5	0.592	0.707	0.677	1.967	0.761-5.087	0.163
MAGED4	0.582		0.868	2.370	0.873-6.435	0.090
DPYSL3	0.591		0.569	1.767	0.704-4.433	0.225
AJAP1	0.578		0.551	1.735	0.682-4.414	0.247

HR, hazard ratio; CI, confidence interval.

Supplementary Table 3: Prognostic factors in the validation set

Variables	Univariate			Multivariable		
	Hazard Ratio	95% CI	*P*	Hazard Ratio	95% CI	*P*
Age (≥ 65)	1.38	0.74 – 2.65	0.310			
Gender (male)	2.01	0.73 – 8.34	0.199			
Background liver (cirrhosis)	1.10	0.57 – 2.07	0.769			
Pugh-Child's classification (B)	1.05	0.32 – 6.48	0.944			
AFP (> 20 ng/ml)	1.27	0.68 – 2.37	0.449			
PIVKA II (> 40 mAU/ml)	1.95	1.03 – 3.87	0.042	1.48	0.74 – 3.07	0.270
Tumor multiplicity (multiple)	2.11	1.03 – 4.05	0.042	1.07	0.36 – 3.29	0.907
Tumor size (≥ 3.0 cm)	1.52	0.79 – 3.07	0.209			
Tumor differentiation (well)	0.65	0.28 – 1.34	0.254			
Growth type (invasive growth)	1.80	0.84 – 3.56	0.126			
Serosal infiltration	3.87	2.04 – 7.25	< 0.001	3.63	1.81 – 7.25	< 0.001
Formation of capsule	1.20	0.62 – 2.44	0.599			
Infiltration to capsule	1.55	0.83 – 3.00	0.175			
Septum formation	0.98	0.53 – 1.89	0.959			
Vascular invasion	2.30	1.16 – 4.35	0.018	1.04	0.31 – 3.45	0.951
UICC pathological stage (II/III)	2.54	1.35 – 4.76	0.004	1.23	0.28 – 5.23	0.776
Expression grade (3)	3.17	1.68 – 5.99	< 0.001	2.83	1.42 – 5.64	0.003

CI, confidence interval; AFP, alpha-fetoprotein; PIVKA, protein induced by vitamin K antagonists; UICC, Union for International Cancer Control.

Supplementary Table 4: Primers used in the study

Gene	Type	Sequence (5´ - 3´)	Product size
PRMT5	forward	TCTCATGGTTTCCCATCCTC	102 bp
	reverse	CCTTCTTGGAATTGCTGCAT	
NRAGE	forward	GATTCCCTCAGACCTTTGC	170 bp
	reverse	GAAGGAATCTGAGGCTTCAG	
MAGED2	forward	TAGAGAAGGCAGACGCATCC	110 bp
	reverse	AAGCGAGTTAGACCTGCACC	
MAGED4	forward	GGCGATCTGAGGAAGCTCAT	91 bp
	reverse	CATACTCAGGTGGGTTGCTGT	
PDSS2	forward	GAATCAGGTAGTGTCAGAGG	181 bp
	reverse	GAGGCTATTCCAGCTGTCATG	
SAMSN1	forward	TGCTCAAGAGAAAGCCATCC	97 bp
	reverse	TTATTCCGAAAACGATCGAAA	
KAL1	forward	AACAATGGTTCCCTGGTTTG	110 bp
	reverse	TCACAAAAGCTTTGGCACTG	
DPYSL3	forward	AGAAGAAGGAGGGAGGGAGC	110 bp
	reverse	CTCCCTTGATAAGGAGACGG	
DENND2D	forward	CACTGCTCTACCCCTTCAGC	204 bp
	reverse	TTTTTCATCACCAACCGACA	
AJAP1	forward	GTTAGCACAACGGAGCCTTC	104 bp
	reverse	GATGATCTGATGGACAGCCA	
BTG1	forward	CTGCAGACCTTCAGCCAGA	104 bp
	reverse	CGAATACAACGGTAACCCGA	
GPR155	forward	AGCAAAGCTGGACTATTCCCT	125 bp
	reverse	GCCACCAAATAAATGTACTGGA	
GAPDH	forward	GAAGGTGAAGGTCGGAGTC	226 bp
	probe	CAAGCTTCCCGTTCTCAGCC	
	reverse	GAAGATGGTGATGGGATTTC	
